# Low-Frequency Ventilation May Facilitate Weaning in Acute Respiratory Distress Syndrome Treated with Extracorporeal Membrane Oxygenation: A Randomized Controlled Trial

**DOI:** 10.3390/jcm13175094

**Published:** 2024-08-27

**Authors:** Martina Hermann, Sebastian König, Daniel Laxar, Christoph Krall, Felix Kraft, Katharina Krenn, Clemens Baumgartner, Verena Tretter, Mathias Maleczek, Alexander Hermann, Melanie Fraunschiel, Roman Ullrich

**Affiliations:** 1 Department of Anaesthesia, General Intensive Care and Pain Medicine, Medical University of Vienna, Spitalgasse 23, 1090 Vienna, Austria; martina.hermann@meduniwien.ac.at (M.H.); sebastian.koenig@meduniwien.ac.at (S.K.); felix.kraft@meduniwien.ac.at (F.K.); eva.tretter@meduniwien.ac.at (V.T.); mathias.maleczek@meduniwien.ac.at (M.M.); 2 Ludwig Boltzmann Institute for Digital Health and Patient Safety, Währingerstraße 104/10, 1180 Vienna, Austria; daniel.laxar@dhps.lbg.ac.at; 3 Center for Medical Statistics, Informatics and Intelligent Systems, Medical University of Vienna, 1090 Vienna, Austria; christoph.krall@meduniwien.ac.at; 4 Department of Internal Medicine III, Division of Endocrinology and Metabolism, Medical University of Vienna, Spitalgasse 23, 1090 Vienna, Austria; clemens.baumgartner@meduniwien.ac.at; 5 Department of Medicine I, Intensive Care Unit 13i2, Medical University of Vienna, Spitalgasse 23, 1090 Vienna, Austria; alexander.hermann@meduniwien.ac.at; 6 IT4Science, Medical University of Vienna, Spitalgasse 23, 1090 Vienna, Austria; melanie.fraunschiel@meduniwien.ac.at; 7 Department of Anesthesiology and Intensive Care Medicine, AUVA Trauma Center Vienna, Kundratstraße 37, 1120 Vienna, Austria

**Keywords:** acute respiratory distress syndrome, extracorporeal membrane oxygenation, COVID-19, invasive mechanical ventilation, low-frequency ventilation, ventilator-free days

## Abstract

Although extracorporeal membrane ventilation offers the possibility for low-frequency ventilation, protocols commonly used in patients with acute respiratory distress syndrome (ARDS) and treated with extracorporeal membrane oxygenation (ECMO) vary largely. Whether strict adherence to low-frequency ventilation offers benefit on important outcome measures is poorly understood. **Background/Objectives**: This pilot clinical study investigated the efficacy of low-frequency ventilation on ventilator-free days (VFDs) in patients suffering from ARDS who were treated with ECMO therapy. **Methods**: In this single-center randomized controlled trial, 44 (70% male) successive ARDS patients treated with ECMO (aged 56 ± 12 years, SAPS III 64 (SD ± 14)) were randomly assigned 1:1 to the control group (conventional ventilation) or the treatment group (low-frequency ventilation during first 72 h on ECMO: respiratory rate 4–5/min; PEEP 14–16 cm H_2_O; plateau pressure 23–25 cm H_2_O, tidal volume: <4 mL/kg). The primary endpoint was VFDs at day 28 after starting ECMO treatment. The major secondary endpoint was ICU mortality, 28-day mortality and 90-day mortality. **Results:** Twenty-three (52%) patients were successfully weaned from ECMO and were discharged from the intensive care unit (ICU). Twelve patients in the treatment group and five patients in the control group showed more than one VFD at day 28 of ECMO treatment. VFDs were 3.0 (SD ± 5.5) days in the control group and 5.4 (SD ± 6) days in the treatment group (*p* = 0.117). Until day 28 of ECMO initiation, patients in the treatment group could be successfully weaned off of the ventilator more often (OR of 0.164 of 0 VFDs at day 28 after ECMO start; 95% CI 0.036–0.758; *p* = 0.021). ICU mortality did not differ significantly (36% in treatment group and 59% in control group; *p* = 0.227). **Conclusions**: Low-frequency ventilation is comparable to conventional protective ventilation in patients with ARDS who have been treated with ECMO. However, low-frequency ventilation may support weaning from invasive mechanical ventilation in patients suffering from ARDS and treated with ECMO therapy.

## 1. Introduction

Acute respiratory distress syndrome (ARDS) can be caused by a variety of underlying conditions and frequently requires highly invasive mechanical ventilation (IMV) [1]. Application of high-plateau pressure levels or large tidal volumes in patients with ARDS may harm the lungs independently, a phenomenon referred to as ventilator-induced lung injury (VILI). Involved pathomechanisms include barotrauma, atelectotrauma and volutrauma due to alveolar overdistension and biotrauma due to an excessive inflammatory response [2,3]. Limiting plateau and driving pressures as well as tidal volumes (TV) has been shown to reduce mortality [4,5,6]. Aside from prone positioning, muscle relaxation, high-dose steroids, recruitment maneuvers and nitric oxide (NO) inhalation, extracorporeal membrane oxygenation (ECMO) serves as an “ultima ratio” intervention in patients with severe ARDS and refractory hypoxemia [7,8,9]. During the coronavirus disease 2019 (COVID-19) pandemic, ECMO globally gained importance beyond rescue situations. Irrespective of the cause for ARDS, there are no clear recommendations for mechanical ventilation strategies for patients on ECMO [10,11]. ECMO permits a wide range of possible ventilation approaches, including the application of extremely low TVs and pressure limits. Apart from some promising results from preclinical studies, it remains undefined whether approaches with ultra-low invasiveness are superior to conventional protective ventilation in the clinical setting. Interleukin-6 (IL-6) appears to be the key circulating cytokine associated with worse outcomes in COVID-19-related ARDS and predicts respiratory insufficiency significantly earlier than C-reactive protein (CRP) [12,13,14]. Therefore, we investigated the safety and efficacy of low-frequency ventilation in patients receiving ECMO therapy for ARDS in a randomized clinical trial.

## 2. Materials and Methods

### 2.1. Study Design

In this randomized controlled clinical trial, we enrolled mechanically ventilated patients suffering from ARDS and treated with ECMO. Study sites were the Department of Anaesthesia, General Intensive Care and Pain Medicine and the Department of Medicine I of the Medical University of Vienna.

### 2.2. Inclusion Criteria and Randomization

Patients were enrolled if their medical condition fulfilled the Berlin definition for ARDS [15] and the decision for ECMO treatment was made by treating physicians who were not involved in the conduct of this study. Inclusion criteria were defined as moderate to severe ARDS (PaO_2_/FiO_2_ < 200 mmHg) before the start of ECMO, ECMO < 24 h in situ and age >18 years. Exclusion criteria were defined as BMI > 40 kg/m^2^, expected weaning of ECMO < 3 days, combustion, restrictive chest wall impairment and pregnancy. If patients fulfilled all inclusion criteria and no exclusion criteria occurred within 24 h after initiation of ECMO, we assigned patients 1:1 either to treatment or control group by using block randomization provided to the Medical University of Vienna by the Randomization Service for Multicenter Clinical Trials (Randomizer Version 2.1.0, Institute for Medical Informatics, Statistics and Documentation, Medical University of Graz). Trial Registration: https://clinicaltrials.gov/, NCT03764319.

### 2.3. Interventions

The applied ventilation strategies of the two different groups are summarized in Table 1. Within 24 h after randomization to the treatment group, IMV was adjusted to the targeted parameters as indicated in Table 1. The duration of the study intervention in the treatment group was 72 h from inclusion. From recorded ventilation settings, we calculated driving pressure (∆P) and mechanical power (MP). We calculated MP by using the simplified equation from Becher et al. [16]. In short, MP was calculated as MP = 0.098 · RR · TV · (driving pressure + PEEP). The driving pressure was calculated plateau pressure minus PEEP (all included patients received a pressure-controlled ventilatory mode).

**Figure 1:**
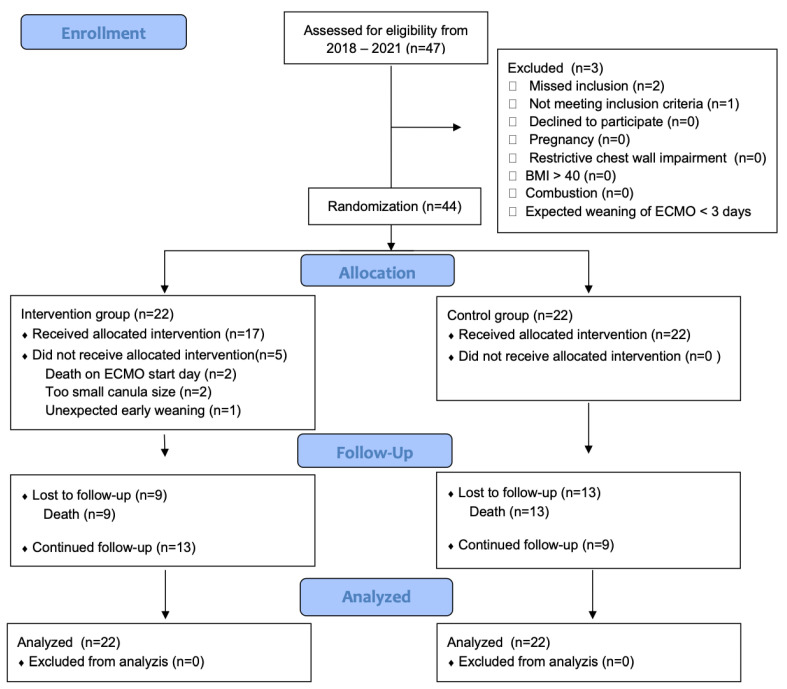
Consolidated standards of reporting trials flow diagram. BMI = body mass index; ECMO = extracorporeal membrane oxygenation.

When patients were randomized to control group, ventilation was continued according to the treating clinicians’ individual decision and based on local recommendations (see Table 1 and Figure 1, Consolidated standards of reporting trials flow diagram).

Data sources

Assessed data included baseline demographics and physiological parameters prior to ECMO initiation, as well as clinical (including need for vasopressor therapy and acute kidney injury with renal replacement therapy) and laboratory parameters during the ICU stay. Simplified acute physiology score III (SAPS III) [17] was calculated at ICU admission. Immunosuppression was defined according to the IDEA Study Group protocol [18]. Patient identification and data collection were conducted using the patient data management system’s routine documentation (ICCA©, Philips, Amsterdam, Netherlands). Electronic data were recorded in the data management software “Clincase”, version 2.7.0.12 (Quadratek Data Solutions Limited, Münzstraße 15, 10,178 Berlin, Germany), hosted by the IT Services & strategisches Informationsmanagement, Medical University of Vienna, 1090 Vienna, Austria. The electronic case report form was designed by the science support work group “IT4Science”. Clincase provided advanced data management and monitoring, maintaining the GCP criteria. Accessible from multiple devices and locations, the web-based electronic case report form allows efficient user handling and moreover error avoidance and data preparation for statistical evaluation during or after the trial. All blood samples were processed and stored at the Medical University Vienna Biobank at the Department of Laboratory Medicine [19]. Analyses of routine laboratory parameters were performed at the Central Clinical Laboratory of the Medical University of Vienna. Additional laboratory parameters for the assessment of inflammation, including serum IL-6, were analyzed in the research laboratory of the Department of Anaesthesia, General Intensive Care and Pain Medicine using the Luminex Assay (Bio-techne; Minneapolis, MN, USA) and analyzed on a MAGPIX analyzer. The datasets supporting the conclusions of this article are included within the Supplementary Materials.

Endpoints/Aims

The primary endpoint was ventilator-free days (VFDs) until day 28 after the start of ECMO. VFDs were defined as days free from mechanical ventilation. In intubated or tracheotomized patients, a T-piece trial or application of continuous positive airway pressure with pressure support <5 cm H_2_O were considered as unassisted breathing techniques, and the respective days were deemed ventilator-free. In case of death during mechanical ventilation, VFD was reported as 0 [20]. The main secondary endpoint was ICU mortality, followed by 28-day and 90-day mortality. Additional endpoints included ECMO duration, ICU length of stay (LOS) and serum concentrations of IL-6 during the intervention period of 72 h.

Statistical methods

Normally distributed metric variables are reported as either means with standard-deviation (SD) or medians (25th–75th percentile). Categorical variables are given as absolute and relative frequencies and either the Chi-squared test or Fisher’s exact test was used for comparison between groups. To determine the effect of the low-frequency ventilation in the treatment group on probability and time of weaning from ventilation, a zero-inflated Poisson regression of VFDs was performed, with SAPS III and covariates showing an effect on VFDs. ICU mortality was analyzed with logistic regression. To investigate the effects of treatment on IL-6 levels over the whole investigation period of 28 days, a mixed linear model with the fixed effects day, group and interaction and the random effect intercept per patient was analyzed for logarithmically transformed values of IL-6. We considered *p* values < 0.05 as statistically significant. Since no multiplicity corrections were applied, *p* values from secondary analyses serve only descriptive purposes. Calculations were performed using R statistics software (version 4.0.5, The R Foundation for Statistical Computing, Vienna, Austria).

## 3. Results

This study was designed as a safety and proof-of-concept study. Between November 2018 and January 2021, we identified 47 consecutive patients admitted to our ICUs who required treatment with ECMO for ARDS. Of these patients, 44 could be included in the study and were randomized 1:1 to either the treatment or control group (n = 22 each). Twenty-six (59%) patients suffered from ARDS due to COVID-19 (treatment group n = 14 (64%), control group n = 12 (55%)). Sixteen (89%) out of eighteen patients suffered from non-COVID-19 ARDS. Of these, the majority (88%) had an underlying pulmonary pathology, such as viral pneumonia (e.g., influenza A), aspiration pneumonia and interstitial lung diseases, among others. The mean age was 56 (SD ± 12) years. Thirty-one patients (70%) were male, the mean pre-ECMO SAPS III was 64 (SD ± 14) and the mean BMI was 30 (SD ± 9) kg/m^2^. Demographic data and detailed baseline information are depicted in Table 2.

Prior to ECMO start (baseline), the mean PEEP was 12 (SD ± 3) cm H_2_O, driving pressure was 17 (SD ± 5) cm H_2_O and median MP was 18.6 (IQR 9.6,28.9) J/min. The arterial blood gas analysis prior to ECMO showed a mean PaO_2_/FiO_2_ ratio of 96 (SD ± 62) mmHg, a mean PaCO_2_ of 71 (SD ± 27) mmHg and a mean pH of 7.29 (SD ± 0.13). Baseline laboratory values are shown in Table S1.

**Main findings**

**Figure 2:**
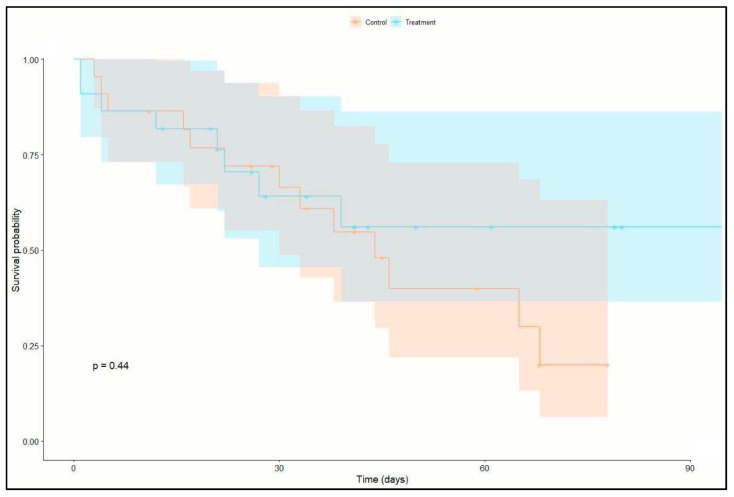
Kaplan–Meier survival analysis.

Twenty-three patients (52%) were both successfully weaned from ECMO and discharged from the ICU. Of these 23 patients, 17 patients had >1 VFDs at day 28 of ICU stay (12 patients in the treatment group and 5 patients in the control group). Twenty-one patients (47%) died in the ICU (8 patients in the treatment group and 13 in the control group). No difference in survival could be identified between the two groups (*p* = 0.227). Kaplan–Meier survival analysis shows a nonsignificant higher survival probability within the treatment group (*p* = 0.44). Please see Figure 2 (Kaplan–Meier survival analysis).

**Figure 3:**
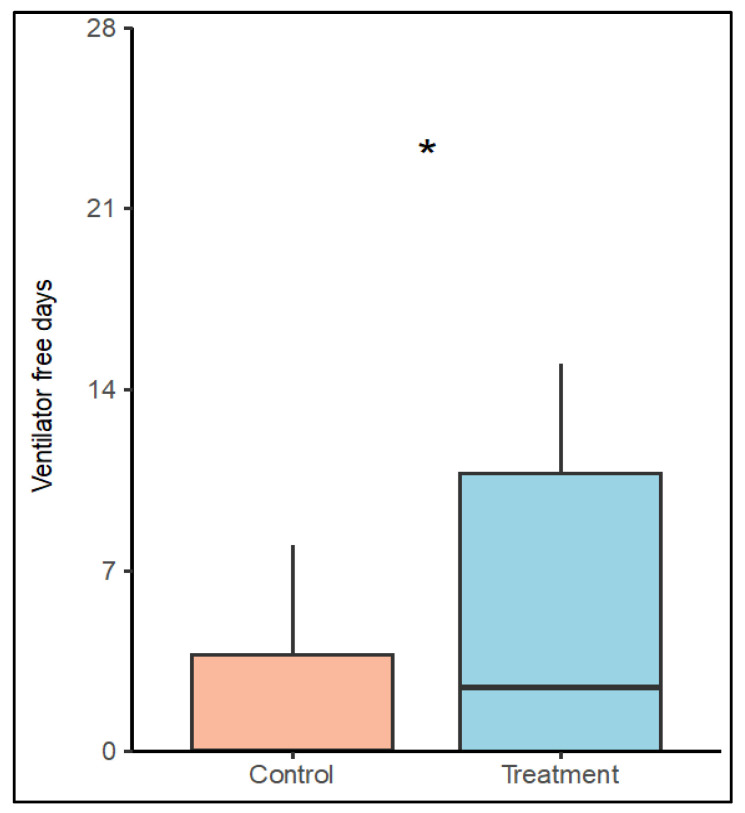
Boxplot VFDs until day 28 versus groups. Asterisk (*) indicates a significantly higher chance for VFDs in a multiple zero-inflated Poisson regression model including treatment group, SAPS III and sex on ventilator-free days (*p* = 0.021). SAPS III = simplified acute physiology score.

The number of VFDs was 3.0 (SD ± 5.5) days in the control group and 5.4 (SD ± 6) days in the treatment group (*p* = 0.117). Simple zero-inflated Poisson regression models identified a positive effect of treatment on the probability for weaning (*p* = 0.07) and a negative effect of male sex on the number of VFDs (*p* = 0.077). Patients in the treatment group showed a significantly higher chance of being weaned from the ventilator after ECMO start until day 28 (*p* = 0.021). Weaning failure was associated with increased SAPS III (*p* = 0.030). In a model including the treatment group, sex and SAPS III as covariates, a positive effect of low-frequency ventilation (OR: 0.16, 95% C.I.: [0.04, 0.76], *p* = 0.021) and a negative effect of SAPS III (OR: 1.07, 95% C.I.: [1.01, 1.013], *p* = 0.03) were identified on the number of VFDs. See Figure 3 (Boxplot VFDs until day 28 versus groups) and Table S2.

Table S3 shows OR for VFDs. OR for 0 VFDs was lower (OR: 0.312, 95% C.I.: [0.089, 1.1], *p* = 0.07) in the treatment group than in the control group (*p* = 0.07). No differences in VFDs between patients with and without COVID-19 could be identified (OR: 2.25, 95% C.I.: [0.65, 7.81, *p* = 0.201]; RR: 0.901, 95% C.I.: [0.667, 01.218], *p* = 0.123). When 28-day mortality was adjusted for the treatment group, SAPS III and sex, there was no effect of either category.

**Figure 4:**
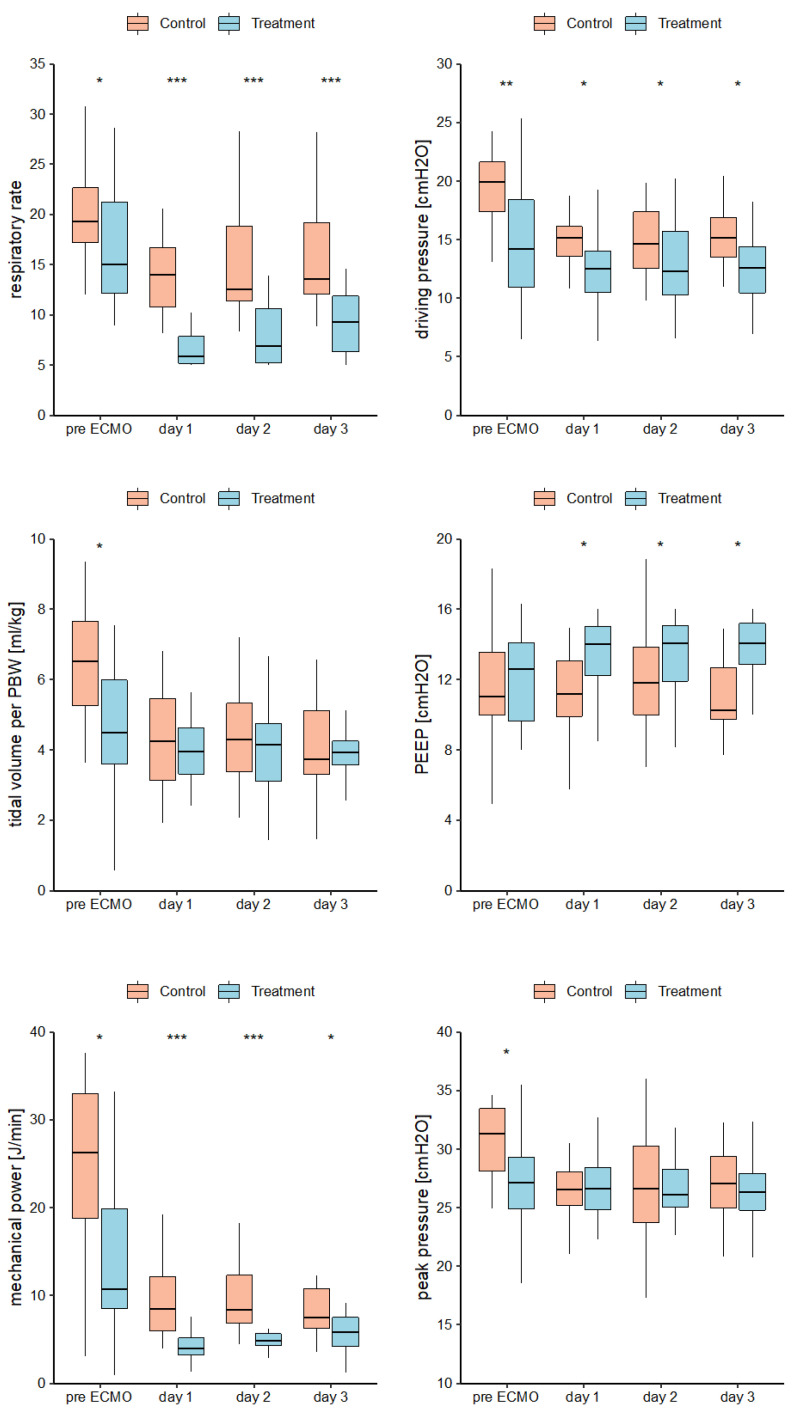
Mean mechanical ventilation parameters versus groups per days pre-ECMO and days 1–3 on ECMO. Asterisk indicates significant differences between groups (*** *p* = < 0.001, ** *p* = 0.001, * *p* = < 0.05). PBW = predicted body weight. PBW female = 45.5 + 0.9 x (height [cm]—152); PBW male = 50 + 0.9 x (height [cm]—152); PEEP = positive end expiratory pressure; mechanical power = 0.098 × respiratory rate x tidal volume (l) × (driving pressure + PEEP).

Median pre-ECMO IMV duration was 2.5 (IQR 1,8) days in the control group and 7.5 [5.2,10] days in the treatment group (*p* = 0.015). On day 1 (the first day on ECMO), RR was significantly higher in the control group as compared to treatment group (14 ± 4 vs. 7 ± 3). PEEP was lower in control group 12 (SD ± 3) than in treatment group 13 (SD ± 2). The mean ∆P was 15 (SD ± 2) in the control group and 13 (SD ± 4) in the treatment group. The median MP was 8.5 (IQR 6, 12.1) in the control group and 3.9 [IQR 3.2, 5.2]) in the treatment group. These differences were identified using two-sided t-tests (RR: *p* < 0.001, PEEP: *p* = 0.03, MP: *p* < 0.001) and a Wilcoxon test (∆P: *p* = 0.031). Groups did not differ for TV, TV per PBW and peak pressures. See Table S4 and Figure 4 (Mean mechanical ventilation parameters versus groups per days pre-ECMO and days 1–3 on ECMO), respectively.

Details of ECMO-related data are shown in Table 3. Thirty-eight patients (86%) underwent treatment with VV ECMO, and six patients (14%) with VA ECMO. The median ECMO duration was 13 (IQR 7,27) days, with a maximum of 76 days. No difference could be identified for ECMO duration between the two groups (*p* = 0.372).

IL-6 parameters are shown in Tables S5 and S6. None of the parameters differed between groups at baseline. Baseline mean IL-6 levels were similar in control and treatment groups (264 pg/mL ± 279 vs. 745 pg/mL ±1121; *p* = 0.243). No significant changes in IL-6 levels were found during the interventional period. Neither group (OR: 0.57, 95% C.I.: [−0.19,1.32], *p* = 0.15), nor day (OR: 0.02, 95% C.I.: [0,0.04], *p* = 0.053) had overall time dependent effects of treatment on IL-6 levels over the investigation period of 28 days. However, a negative correlation between day and treatment was identified, indicating a stronger decline of IL-6 levels in treatment patients (OR: −0.06, 95% C.I.: [−0.08, −0.03], *p* = <0.001), as shown in Table S7.

**Outcome data**

The number of VFDs until day 28 on ICU was 3.0 (SD ± 5.5) days in the control group and 5.4 (SD ± 6) days in the treatment group (*p* = 0.117). ICU mortality was 36% in the treatment group and 59% in the control group, without reaching statistical significance (*p* = 0.227). The median ICU length of stay (LOS) was 30 (IQR 17,45) days, without differences between groups (*p* = 0.526). Pre-ECMO IMV duration was not associated with ICU mortality within our study population (*p* = 0.997), as shown in Table 4. Causes of death were multiorgan failure in seven patients (16%), intracranial bleeding in two patients (5%), heart failure in two patients (5%) and unresolved COVID-19 ARDS in three patients (7%). Adverse events are shown in Table S8.

## 4. Discussion

In this randomized controlled clinical trial, in patients treated with ECMO due to moderate or severe ARDS, 72 h of low-frequency ventilation as compared with standard-of-care protective ventilation had no significant effect on VFDs (5.4 days versus 3 days (*p* = 0.117)). However, our findings show that patients treated with low-frequency ventilation had higher rates of successful weaning from the ventilator (i.e., successful extubation) until day 28 (OR: 0.16, 95% C.I.: [0.04, 0.76], *p* = 0.021). These findings suggest that a low-frequency ventilation strategy could potentially cause less lung damage, thus shortening the need for IMV in patients treated with ECMO.

An experimental animal model describing less histologic lung injury but with a similar ventilation strategy as applied in our study also corroborates these findings [21]. Studies in humans also suggest that decreased ventilation pressures reduce pulmonary biotrauma and thus VILI. Bein et. al. [3] compared a ventilation strategy of 3 mL/kg PBW combined with arteriovenous extracorporeal CO_2_ elimination (avECCO2-R) (low TV group) to 6 mL/kg PBW without avECCO2-R (“normal” lung-protective management) and demonstrated significantly higher VFDs in the low TV group, without showing a significant reduction in ventilation pressures. In our study, low-frequency ventilation reached a marked reduction in ventilation pressures and MP as compared to the control group within the first three days of ECMO treatment. In addition, low-frequency ventilation under ECMO was well tolerated and had no measurable side effects.

Therefore, our study suggests considering the concept of low-frequency ventilation as a valid option for patients on ECMO [22,23]. In our study, mortality did not differ between groups. The Kaplan–Meier survival analysis showed a nonsignificant higher survival probability within the treatment group within 90-day mortality (*p* = 0.44). This is in accordance with recent studies that have failed to show a beneficial effect of low-frequency ventilation on survival [24]. Side effects of ultraprotective ventilation strategies could contribute to adverse outcomes, thus counteracting the beneficial effects of low-frequency ventilation on VILI. In addition, patient–ventilator asynchronies and permissive hypercapnia may be more common in low-frequency ventilation [25,26] due to the marked reduction in respiratory rate, leading to an increased need for sedation [27] and muscle relaxation. These unwanted effects may contribute to protracted weaning and may induce further lung damage due to self-inflicted lung injury in patients.

Additional side effects of low-frequency ventilation include insufficient arterial oxygenation or the removal of carbon dioxide, necessitating the application of higher tidal volumes or respiratory rates. This may provide an explanation for why some patients did not reach target values of RR, PEEP, peak pressure and TV in the treatment group. However, RR and MP were overall markedly lower in the treatment group as compared to control, confirming the targeted minimum ventilation invasiveness. Partial incompliance with our low-frequency ventilation targets can also be explained by spontaneous breathing efforts of patients since the use of neuromuscular blocking agents was at the discretion of the treating ICU physicians and not defined in our study protocol.

When patients are ventilated with very low tidal volumes, both atelectasis and regional overdistension of the ventilated lung may occur [22]. However, the potential for baro- and volutrauma is significantly decreased with a low-frequency ventilation strategy. Further, atelectrauma can be addressed with adequate PEEP settings. Although studies have shown an independent association of ∆P on mortality [2,10], a conventional lung-protective ventilation strategy using a TV of 6 mL/kg PBW and higher levels of PEEP does not support reducing ∆P below 15 cm H_2_O, as used, for example, in the EOLIA trial [2,28,29].

Moreover, ECMO is an invasive technique that carries serious risks, including technical difficulties during cannula insertion as well as required systemic anticoagulation and large bore vessel access (required for high blood flows), all of which contribute to an increased risk of bleeding, a main contributor to ECMO-related morbidity and mortality. The decision on cannula size, configuration mode and time of ECMO start was determined by the treating physicians independently from and prior to study randomization. This liberal approach may explain why the target values of the ventilation settings were not reached in all patients in the experimental treatment group. The ECMO blood flow and sweep gas flow did not differ between the groups.

About half of the patients suffered from ARDS due to COVID-19 pneumonia. COVID-19 can be considered as a unique entity of disease with specific pathological mechanisms, which may alter the disease course as compared to other ARDS etiologies. However, the distribution of COVID-19 patients was similar between groups. Consequently, when we analyzed the VFDs, no difference was found between patients with and without COVID-19-related ARDS. The overall ICU mortality was 48% and did not differ between COVID-19-negative and COVID-19-positive patients. This finding agrees with previous studies of COVID-19-related ARDS and severe ARDS from other causes that were treated with ECMO [30,31]. Due to the heterogeneity of our study population, our treatment regime might be applicable even for a different patient group suffering from pulmonary insufficiency (e.g., bacterial pneumonia).

The IMV duration prior to ECMO start was twice as long in the treatment group as compared to control group. The potential contribution of pre-ECMO IMV duration on outcome measures in patients treated with ECMO remains controversial, particularly in patients with COVID-19 [32]. In our study, pre-ECMO IMV duration did not affect ICU mortality. However, it has to be emphasized that the longer pre-ECMO IMV duration in the treatment group could account for the lower MV invasiveness.

During the first three days of our study period, IL-6 levels did not differ between the two groups. However, a significant decrease in IL-6 occurred in the treatment group after day 21. These findings should be interpreted with caution due to missing values. An experimental study by Retamal et al. described the physiological inflammatory consequences of altered RR [33]. Rozencwajg et al. already observed a significant difference in IL-6 levels (pre-ECMO versus ultraprotective ventilation on ECMO) within seven days, which is much earlier compared to our results [23]. Therefore, our findings may partially be explained by a quicker resolving inflammatory state due to earlier weaning. However, due to the small sample size, these findings should be verified in a larger cohort of patients.

It is well accepted that VILI causes further inflammation and an increase in proinflammatory cytokines such as IL-6 [22]. Elevated levels of these cytokines in patients suffering from ARDS are major determinants of the outcome [34] since they may cause further organ damage, mediating vascular leakage, tissue edema, cellular hypoxia and necrosis. In severely dysregulated immune responses, a cytokine storm can lead to life-threating conditions with multiorgan failure and death [35]. Recently, classification of ARDS into two distinct phenotypes, a hyperinflammatory and a hypoinflammatory phenotype, has been shown to explain the often different responses to therapeutic interventions in clinical trials. A distinction of phenotype follows the severity of systemic inflammation and concentrations of proinflammatory biomarkers, as well as the need for vasopressors and the prevalence of sepsis [36]. The application of these classifiers in patients suffering from ARDS due to COVID-19 infection failed to reproduce the findings in non-COVID-19-induced ARDS. Several studies have suggested that endothelial dysfunction may be more important in COVID-19 ARDS as compared to non-COVID-19 ARDS caused by pneumonia [37,38]. The hypothesis that mortality in COVID-19 is due to an exaggerated systemic inflammatory response is currently not supported by data [39].

**Limitations**

One major limitation of this study was the lack of a strict protocol for use of NMBA to commit interference with low-frequency ventilation through spontaneous breathing efforts during ECMO support in the treatment group. This might explain the nonsignificant difference between groups within the ventilation parameters. Following studies should take particular focus on this aspect.

RR and MP were markedly reduced in the treatment group during the first 72 h, although not all target values for mechanical ventilation settings were consistently achieved. TV and peak pressure did not differ between groups, demonstrating a well-established use of lung-protective ventilation in our clinical routine [40]. However, ventilation invasiveness, expressed as RR, MP, driving pressure, peak pressure and TV, was significantly higher in the control group pre-ECMO. This unintended group disparity might have influenced our primary endpoint results. Although the results only suggested a trend towards lower VILI in the treatment group, this disparity could be a significant factor; thus, it serves as a considerable limitation. The still-considerable reduction in RR and MP achieved in the treatment group from pre-ECMO during the following days indicates a marked reduction in overall ventilation invasiveness. This limitation should be addressed in future prospective studies.

IL-6 measurements were not available from all patients. Further, no bronchoalveolar lavage fluid was analyzed for other biomarkers typically associated with VILI to detect differences in regional patterns of the inflammatory response to low-frequency ventilation as compared to conventional lung-protective ventilation.

## 5. Conclusions

In this small clinical trial, 72 h of low-frequency ventilation was compared to conventional protective ventilation in a heterogenous population of patients with ARDS treated with ECMO. The marked reduction in RR and TV led to a significant lowering of driving pressure and MP and did not impair gas exchange. The overall outcomes were similar in both groups, except that low-frequency ventilation may facilitate earlier weaning of patients from the ventilator. This finding suggests that low-frequency ventilation may “rest” the lung and potentially lead to reduced lung damage. Additional studies with larger sample sizes are needed to better define the role of low-frequency ventilation in ARDS patients treated with ECMO.

## Figures and Tables

**Table 1:** Mechanical ventilation settings.

	Control Group	Treatment Group
Duration		72 h from inclusion ^1^
Ventilation Mode	Conventional settings ^2^	Pressure controlled ventilation
Respiratory Rate	12–25 per minute	4–5 per minute
PEEP	≥10 cm H_2_O	14–16 cm H_2_O ^3^
Peak Pressure	<30 cm H_2_O	23–25 cm H_2_O ^4^
Tidal Volume	<6 mL/kg PBW	4 mL/kg PBW
I:E	1:1–1:2	1:5

PEEP = positive end expiratory pressure; PBW = predicted body weight; PBW was calculated as 45.5 + [0.91 (centimeters of height − 152.4)] for females and 50 + [0.91 (centimeters of height − 152.4)] for males; TV = tidal volume; I:E = inspiratory:expiratory time. ^1^ After 72 h, the treatment group was switched to conventional ventilator management according to the treating clinician’s decision; ^2^ Conventional settings applied by the treating physicians according to the recent guidelines of our center; ^3^ PEEP adjusted to achieve a peak pressure of 23–25 cm H_2_O; ^4^ peak pressure adjusted to not exceed TV of 4 mL/kg PBW.

**Table 2:** Demographic data and baseline characteristics, pre-ECMO ventilation parameters, according to their randomization.

	All Patients	Control	Treatment	*p* Value	
Age, mean (SD)–years	56(±12)	56(±10)	56(±14)	0.912	
Sex, Male, no. (%)	31(70)	16(73)	15(68)	1	
BMI, mean (SD)–kg/m^2^	30(±9)	30(±11)	29(±6)	0.738	
COVID-19, no. (%)	26(59)	12(55)	14(64)	0.759	
SAPS III, mean (SD)	64(±14)	60(±14)	67(±13)	0.074	
Tracheostomy, no. (%)	30(68)	18(82)	12(55)	0.106	
**COMORBIDITIES**	**All patients**	**Control**	**Treatment**	***p* value**	
Arterial hypertension, no. (%)	19(43)	9(41)	10(45)	0.851	
Chronic heart disease, no. (%)	6(14)	2(9)	4(18)	0.659	
Obesity, no. (%)	5(11)	3(14)	2(9)	0.690	
Diabetes, no. (%)	8(18)	3(14)	5(23)	0.794	
Chronic respiratory disease, no. (%)	8(18)	3(14)	5(23)	0.672	
Chronic kidney disease, no. (%)	18(41)	10(23)	8(18)	0.759	
Immunosuppression, no. (%)	6(14)	4(18)	2(9)	0.763	
**VENTILATION PRE-ECMO**	**All patients**	**Control**	**Treatment**	***p* value**	
PEEP, mean (SD)–cm H_2_O	12(±3)	11(±3)	12(±3)	0.316	
TV, mean (SD)–ml	355(±141)	401(±144)	309(±126)	0.030	
TV, mean (SD)–ml/kg PBW	5.4(±2.1)	6.2(±2.2)	4.6(±1.9)	0.013	
Respiratory rate, mean (SD)–/min	18(±5)	20(±4)	16(±6)	0.021	
Peak pressure, mean (SD)–cm H_2_O	29(±4)	31(±3)	27(±4)	0.003	
Driving pressure, mean (SD)–cm H_2_O	17(±5)	19(±3)	15(±5)	0.001	
MP, median (IQR)–J/min	18.6(9.6,28.9), n = 43	26.9(18.6,33.2), n = 21	10.7(8.5,19.9), n = 22	0.002	
**BASELINE VALUES PRE-ECMO**	**All patients**	**Control**	**Treatment**	***p* value**	
pre-ECMO IMV, median (IQR)–days	6(1,10)	2.5(1,8)	7.5 (5.2,10)	0.015	
PaO_2_/FiO_2_, mean (SD)	96(±62)	82(±50)	109(±71)	0.324	
PaO_2_, mean (SD)–mmHg	73(±20), n = 40	73(± 19), n = 19	72(±21), n = 21	0.875	
PaCO_2_, mean (SD)–mmHg	71(±27), n = 40	68(±19), n = 19	73(±33), n = 21	0.521	
pH, mean (SD)–mmHg	7.29(±0.13), n = 40	7.31(±0.10), n = 19	7.27(±0.15), n = 21	0.403	
BE, mean (SD)–mmol/L	6(±6), n = 40	6(±6), n = 19	5(±7), n = 21	0.553	
NMBA, no. (%)	20(45%)	11(50%)	9(41%)	0.762	

Metric data are reported as means (± SD) or medians (IQR); n gives the number of available observations. Categorical variables are reported as absolute and relative frequencies and compared between groups using Chi-squared tests or Fisher´s exact tests. BMI = body mass index; COVID 19 = coronavirus disease 2019; SAPS III = simplified acute physiology score; PEEP = positive end expiratory pressure; TV = tidal volume; PBW = predicted body weight. PBW female = 45.5 + 0.9 x (height [cm]—152); PBW male = 50 + 0.9 x (height [cm]—152); MP = mechanical power = 0.098 x respiratory rate x tidal volume (l) x (driving pressure + PEEP); ECMO = extracorporeal membrane oxygenation; IMV = invasive mechanical ventilation; PaO_2_ = partial pressure of arterial oxygen; FiO_2_ = fraction of inspired oxygen; PaCO_2_ = partial pressure of arterial carbon dioxide; BE = base excess; NMBA = neuromuscular blocking agents.

**Table 3:** ECMO configurations during 72 h, according to their randomization.

ECMO CONFIGURATION MEAN DURING 72 h on ECMO	All Patients	Control	Treatment	*p* Value
Venoarterial, no. (%)	6(14%)	1(5%)	5(23%)	0.188
Venovenous, no. (%)	38(86%)	21(95%)	17(77%)	0.188
ECMO blood flow, mean (SD)-l/min	3.2(±0.7)	3.1(±0.6)	3.3(±0.8)	0.373
ECMO sweep gas flow, mean (SD)-l/min	3.9(±1.2)	3.6(±1.3)	4.3(±1.1)	0.066

ECMO = extracorporeal membrane oxygenation.

**Table 4:** Outcome data and cause of death between groups.

OUTCOME	All Patients	Control	Treatment	*p* Value
Ventilator-free days until day 28, mean (SD)–days	4.2(±5.8)	3(±5.5)	5.4(±6)	0.117
ICU LOS, median (IQR)–days	30(17,45)	34(18,46)	27(15,43)	0.526
ECMO duration, median (IQR)–days	13(7,27)	16(8,30)	12(6,23)	0.372
ICU mortality, no. (%)	21(48)	13(59)	8(36)	0.227
28-day mortality, no. (%)	12(27)	5(23)	7(32)	0.735
90-day mortality, no. (%)	17(39)	10(45)	7(32)	0.536
pre-ECMO IMV, mean (SD)–days vs. ICU death	8(±8)	8(±6)	8(±10)	0.997
**CAUSE OF DEATH**	**All patients**	**Control**	**Treatment**	***p* value**
Multiorgan failure, no. (%)	7(16)	4(18)	3(14)	1
Intracranial bleeding, no. (%)	2(5)	2(9)	0(0)	0.57
Heart failure, no. (%)	2(5)	1(5)	1(5)	0.864
COVID-19 ARDS, no. (%)	3(7)	1(5)	2(9)	0.784

ICU = intensive care unit; LOS= length of stay; ECMO = extracorporeal membrane oxygenation; IMV = invasive mechanical ventilation; COVID-19 = coronavirus disease 2019; ARDS = acute respiratory distress syndrome.

## Data Availability

The dataset supporting the conclusions of this article is available and saved at the Department of Anaesthesia, General Intensive Care and Pain Medicine. Data are available from the authors upon reasonable request.

## Supplementary Materials

The following supporting information can be downloaded at: https://www.mdpi.com/article/10.3390/jcm13175094/s1, Table S1. Baseline laboratory values between groups; Table S2. Mechanical ventilation parameters versus groups per days 1–3 on ECMO; Table S3. IL-6 levels between groups during the intervention period of 72 h; Table S4. IL-6 levels of patients with and without COVID-19 during the intervention period of 72 h; Table S5. Multiple zero-inflated Poisson regression model with treatment group, SAPS III and sex on ventilator-free days; Table S6. Simple zero-inflated Poisson regression model for VFD on covariates; Table S7. Linear mixed model with fixed effects of treatment group and days over 28 days; Table S8. Adverse Events, as documented by the attending physicians.
