# Supplementary material for: Low-Frequency Ventilation May Facilitate Weaning in Acute Respiratory Distress Syndrome Treated with Extracorporeal Membrane Oxygenation: A Randomized Controlled Trial
Source: J Clin Med. 2024 Aug 27;13(17):5094. doi: 10.3390/jcm13175094 (PMC11396271; doi:10.3390/jcm13175094)
Supplement: Supplementary file 1 [file jcm-13-05094-s001.zip › jcm-3146429-supplementary.pdf]

Table S1. Baseline laboratory values between groups.

	All patients	Control	Treatment	P value
Platelet Concentrates	1(±3), n=43	2(±5), n=22	0(±1), n=21	0.2
Human albumin Concentrates	19(±36), n=43	16(±19), n=22	22(±49), n=21	0.625
PRBC	14(±24), n=43	17(±32), n=22	10(±10), n=21	0.318
Hemoglobin, mean (SD) - g/dl	10(±2), n=33	10(±2), n=17	10(±1), n=16	0.315
Platelets, mean (SD) - G/l	255(±116), n=32	232(±125), n=16	279(±105), n=16	0.258
Leukocytes, mean (SD) - G/l	14(±7), n=33	14(±8), n=17	13(±5), n=16	0.784
PTT, mean (SD) - s	46(±22), n=32	48(±29), n=17	43(±9), n=15	0.535
Fibrinogen, mean (SD) – mg/dL	623(±262), n=32	593(±299), n=17	657(±217), n=15	0.489
C-reactive protein, mean (SD) – mg/dL	18(±11), n=32	16(±11), n=17	19(±12), n=15	0.445
Interleukin-6, mean (SD) – pg/mL	504(±831), n=18	264(±279), n=9	745(±1121), n=9	0.243
Procalcitonin, mean (SD) – ng/mL	1(±1), n=17	1(±1), n=8	1(±1), n=9	0.386
Creatinine, mean (SD) – mg/dl	1.12 (±0.67), n=32	1.08(±0.6), n=17	1.16(±0.75), n=15	0.741
Urea, mean (SD) – mg/dl	31(±13), n=32	29(±12), n=17	33(±14), n=15	0.407
Sodium, mean (SD) – mmol/L	144(±5), n=40	144(±4), n=19	145(±5), n=21	0.458
Potassium, mean (SD) – mmol/L	5(±1), n=40	5(±1), n=19	5(±1), n=21	0.56
Chloride, mean (SD) – mmol/L	105(±6), n=40	105(±6), n=19	106(±5), n=21	0.496
Calcium, mean (SD) – mmol/L	2.09(±0.21), n=32	2.13(±0.24), n=17	2.06(±0.16), n=15	0.317
Calcium, ionized, mean (SD) – mmol/L	1.17(±0.1), n=40	1.17(±0.1), n=19	1.16(±0.1), n=21	0.691
Magnesium, mean (SD) – mmol/L	1(±0), n=32	1(±0), n=17	1(±0), n=15	0.728
ProBNP, mean (SD) -pg/mL	2607(±4880), n=18	1257(±1182), n=8	3688(±6401), n=10	0.268
Albumin, mean (SD) – g/L	27(±6), n=32	27(±7), n=17	28(±6), n=15	0.658
GOT, median (IQR) – U/L	44[28,63.8] n=32	38[24,49] n=17	57[32,74] n=15	0.157
GPT, median (IQR) – U/L	40[22,50.5] n=32	40[22,49] n=17	46[28.5,104.5] n=15	0.3166
Gamma-GT, median (IQR) -U/L	205[64.2,400] n=32	200[58,321] n=17	210[78.5,464] n=15	0.355
LDH , median (IQR) – U/L	370[296,434] n=33	340[278,430] n=17	383[307,459] n=16	0.461
Bilirubin total, median (IQR) -mg/dl	0.6[0.4,1] n=32	0.6[0.4,1.3] n=17	0.5[0.4,0.9] n=15	0.66
Creatinine kinase, median (IQR) – U/L	103[63,186] n=32	78[58,142] n=17	155[97,223] n=15	0.1

Metric data are reported by mean (± SD) or by median (IQR), n gives the number of available observations. Categorical variables are reported by absolute and relative frequencies and compared by Chi-squared tests or Fisher´s exact tests between groups; PRBC=Packed red blood cells; PTT=Partial Thromboplastin Time; INR=International Normalized Ratio; ProBNP=pro brain natriuretic peptide; GOT=glutamic oxaloacetic transaminase; GPT=glutamic pyruvic transaminase; gamma-GT=gamma-glutamyl transferase; LDH=lactate dehydrogenase.

Table S2. Multiple zero inflated poisson regression model with treatment group, SAPS III and sex on ventilator free days.

covariate	effect	P value
Treatment group: OR (95% CI)	0.164 (0.036,0.758)	0.021
Treatment group: RR (95% CI)	0.825 (0.542,1.258)	0.372
SAPS III: OR (95% CI)	1.065 (1.006,1.126)	0.03
SAPS III: RR (95% CI)	1.01 (0.992,1.028)	0.284
Male: OR (95% CI)	1.729 (0.407,7.338)	0.458
Male: RR (95% CI)	0.752 (0.506,1.117)	0.158

OR=Odds ratio; RR=Risk ratio; SAPS III=simplified acute physiology score.

Table S3. Simple zero inflated poisson regression model for VFD on covariates.

covariate	estimate	P value
Treatment group: OR (95% CI)	0.312 (0.089,1.1)	0.07
Treatment group: RR (95% CI)	0.901 (0.667,1.218)	0.499
COVID-19: OR (95% CI)	2.25 (0.694,7.806)	0.201
COVID-19.: RR (95% CI)	1.256 (0.94,1.679)	0.123
Sex: OR (95% CI)	1.357 (0.367,5.02)	0.647
Sex: RR (95% CI)	0.767 (0.571,1.03)	0.078
Age: OR (95% CI)	1.028 (0.976,1.083)	0.301
Age: RR (95% CI)	1.002 (0.988,1.016)	0.813
BMI: OR (95% CI)	1.025 (0.953,1.102)	0.507
BMI: RR (95% CI)	0.994 (0.967,1.021)	0.652
SAPS III: OR (95% CI)	1.036 (0.989,1.086)	0.134
SAPS III: RR (95% CI)	1.007 (0.994,1.02)	0.287
IMV pre-ECMO: OR (95% CI)	1.017 (0.943,1.098)	0.658
IMV pre-ECMO: RR (95% CI)	0.987 (0.964,1.01)	0.26
PaO2/FiO2: OR (95% CI)	1.005 (0.995,1.016)	0.329
PaO2/FiO2: RR (95% CI)	0.999 (0.996,1.001)	0.288

OR=Odds ratio; RR=Risk ratio; BMI=body mass index; SAPS III=simplified acute physiology score; IMV=invasive mechanical ventilation; PaO2=partial pressure of arterial oxygen; FiO2=fraction of inspired oxygen.

Table S4. Mechanical ventilation parameters versus groups per days 1-3 on ECMO.

VENTILATION DURING ECMO DAY 1	All patients	Control	Treatment	P value
PEEP, mean (SD) – cm H2O	13(±3)	12(±3)	13(±2)	0.03
Tidal volume, mean (SD) – ml	267(±90)	274(±106)	261(±73)	0.644
Tidal volume, mean (SD) – ml/kg PBW	4.1(±1.3)	4.2(±1.5)	3.9(±1.1)	0.439
Respiratory rate, mean (SD) – /min	11(±5)	14(±4)	7(±3)	<0.001
Peak pressure, mean (SD) – cm H2O	26(±3)	27(±3)	26(±3)	0.794
Driving pressure, mean (SD) – cm H2O	14(±3)	15(±2)	13(±4)	0.031
Mechanical power, median [IQR] – J/min	5.7[3.9,9.6]	8.5[6,12.1]	3.9[3.2,5.2]	<0.001
NMBA, no. (%)	26(59)	10(45)	16(73)	0.125

VENTILATION DURING ECMO DAY 2	All patients (n=43)	Control (n=22)	Treatment (n=21)	P value
PEEP, mean (SD) – cm H2O	13(±3)	12(±4)	14 (±2)	0.045
Tidal volume, mean (SD) – ml	273(±92)	279(±101)	267(±83)	0.674
Tidal volume, mean (SD) – ml/kg PBW	4.2(±1.3)	4.3(±1.4)	4.0(±1.3)	0.415
Respiratory rate, mean (SD) – /min	11(±5)	15(±5)	8(±3)	<0.001
Peak pressure, mean (SD) – cm H2O	27(±4)	27(±5)	27(±4)	0.996
Driving pressure, mean (SD) – cm H2O	14(±3)	15(±3)	13(±4)	0.075
Mechanical power, median [IQR] – J/min	5.9[4.7,10.5]	8.4[6.8,12.3]	4.8[4.4,5.6]	<0.001
NMBA, no. (%)	26(60)	12(55)	14(67)	0.617

VENTILATION DURING ECMO DAY 3	All patients (n=41)	Control (n=21)	Treatment (n=20)	P value
PEEP, mean (SD) – cm H2O	12(±3)	11(±4)	14(±2)	0.016
Tidal volume, mean (SD) – ml	258.5(±87)	262(±106)	255(±65)	0.806
Tidal volume, mean (SD) – ml/kg PBW	3.9(±1.2)	4.0(±1.3)	3.8(±0.9)	0.539
Respiratory rate, mean (SD) – /min	12(±5)	15(±5)	9(±3)	<0.001
Peak pressure, mean (SD) – cm H2O	26(±4)	27(±4)	26(±4)	0.733
Driving pressure, mean (SD) – cm H2O	14(±3)	15(±2)	13(±3)	0.003
Mechanical power, median [IQR] – J/min	6.9[4.9,8.8]	7.5[6.3,10.8]	5.9[4.2,7.5]	0.007
NMBA, no. (%)	23(56)	11(52)	12(60)	0.86

ECMO=extracorporeal membrane oxygenation; PEEP=positive end expiratory pressure; PBW=predicted body weight. PBW female = 45.5 + 0.9 * (height [cm] - 152); PBW male = 50 + 0.9 * (height [cm] - 152); mechanical power=0.098 x respiratory rate x tidal volume (l) x (driving pressure + PEEP); NMBA=neuromuscular blocking agents.

Table S5. IL-6 levels between groups during the intervention period of 72 h.

IL-6	All patients	Control	Treatment	P value
Baseline pg/mL, mean (SD)	504(±831), n=18	264(±279), n=9	745(±1121), n=9	0.243
Day 1 pg/mL, mean (SD)	190(±278), n=26	132(±138), n=14	256(±379), n=12	0.301
Day 2 pg/mL, mean (SD)	220(±264), n=24	191(±232), n=13	254(±305), n=11	0.58
Day 3 pg/mL, mean (SD)	152(±158), n=22	88(±46), n=11	215(±204), n=11	0.069

IL-6=Interleukin-6.

Table S6. IL-6 levels of patients with and without COVID-19 during the intervention period of 72h.

IL-6 COVID-19 ARDS	All patients	Non COVID-19	COVID-19	P value
Baseline pg/mL, mean (SD)	504(±831), n=18	29.7(±.), n=1	532(±847), n=11	.
Day 1 pg/mL, mean (SD)	190(±278), n=26	146(±239), n=5	200(±291), n=17	0.676
Day 2 pg/mL, mean (SD)	220(±264), n=24	483(±558), n=5	196(±232), n=16	0.599
Day 3 pg/mL, mean (SD)	152(±158), n=18	13.6(±.), n=4	158(±159), n=14	.

OUTCOME IL-6 COVID-19 ARDS	All patients (n=44)	Non COVID-19 (n=17)	COVID-19 (n=27)	P value
ICU Death, no. (%)	21(48%)	7(41%)	14(52%)	0.227
Ventilator free days till day 28, median (IQR) – days	0[0,9.2]	1[0,9]	0[0,8.5]	0.379

COVID 19=coronavirus disease 2019; IL-6=Interleukin-6; ICU=intensive care unit; IQR=Interquartile Range.

Table S7. Linear mixed model with fixed effects of treatment group and days over 28 days.

IL-6 over 28 days	estimate	P value
Treatment group	0.57 (-0.19,1.32)	0.153
Day	0.02 (0,0.04)	0.053
Treatment group:Day	-0.06 (-0.08,-0.03)	<0.001

IL-6=Interleukin-6

Table S8. Adverse Events, as documented by the attending physicians.

ADVERSE EVENTS DURING 28 DAYS on ICU	All patients	Control	Treatment	P value
Bleeding general	9(20%) n=44	5(23%) n=22	4(18%) n=22	0.079
Gastrointestinal bleeding	2(5%) n=44	0(0%) n=22	2(9%) n=22	0.245
ECMO Bleeding	7(16%) n=44	4(18%) n=22	3(14%) n=22	0.313
Airway Bleeding	13(30%) n=44	8(36%) n=22	5(23%) n=22	0.159
Intracranial bleeding	2(5%) n=44	2(9%) n=22	0(0%) n=22	0.120
Pericardial effusion	5(11%) n=44	3(14%) n=22	2(9%) n=22	0.339
Hemothorax	2(5%) n=44	2(9%) n=22	0(0%) n=22	0.120
Ischemic stroke	20(45%) n=44	12(27%) n=22	8(18%) n=22	0.364
Pulmonary embolism	6(14%) n=44	3(14%) n=22	3(14%) n=22	0.3

ECMO=extracorporeal membrane oxygenation.

Figure 1. Consolidated Standards of Reporting Trials flow diagram.

CONSORT
TRANSPARENT REPORTING of TRIALS

CONSORT Flow Diagram

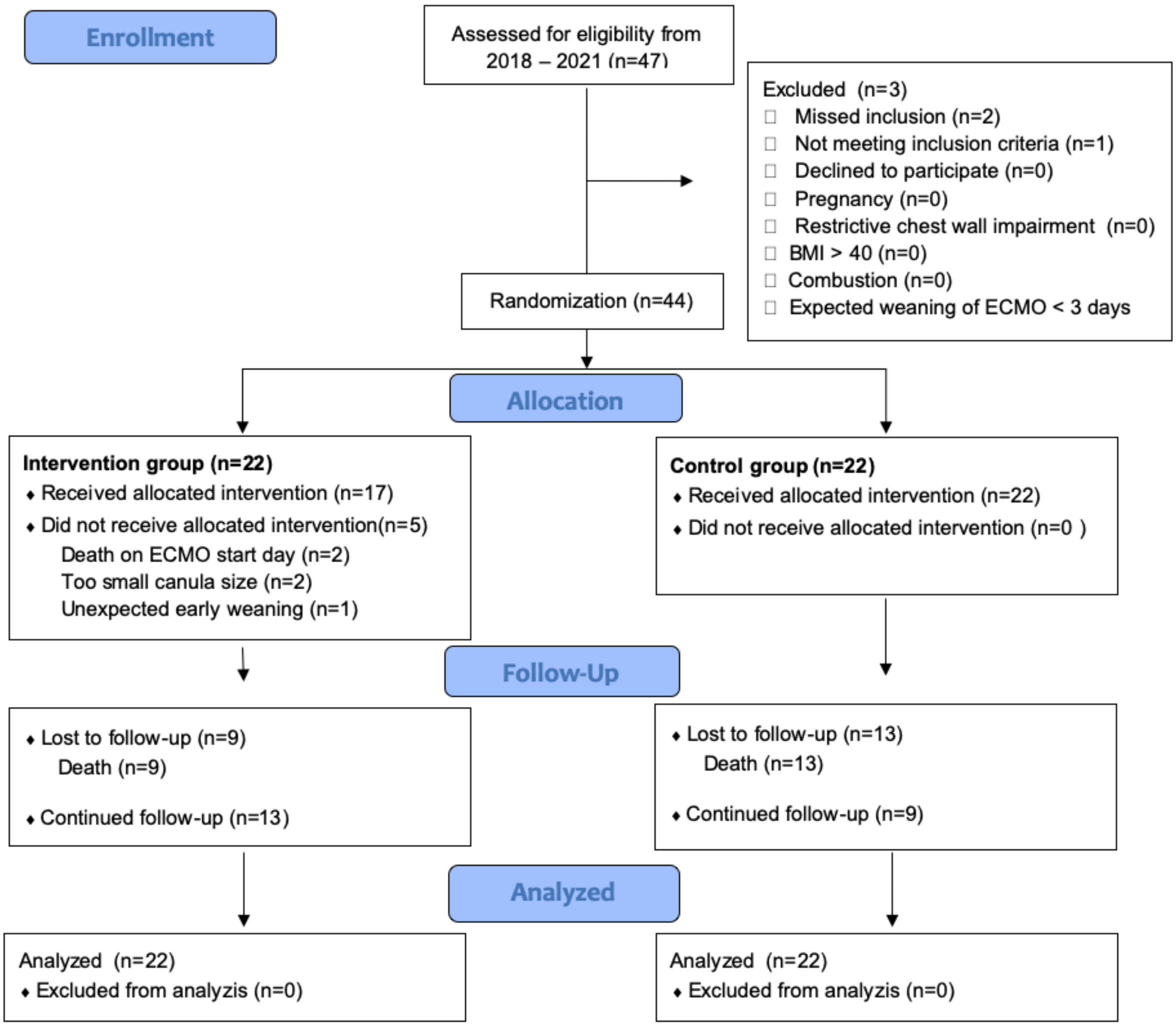

BMI=Body mass index; ECMO=Extracorpreal membrane oxygentation;
